# Whose responsibility is it to talk with children and young people about intersex/differences in sex development? Young people’s, caregivers’ and health professionals’ perspectives

**DOI:** 10.3389/fruro.2023.1089198

**Published:** 2023-03-29

**Authors:** Katrina Roen, Tove Lundberg, Peter Hegarty, Lih-Mei Liao

**Affiliations:** ^1^ School of Social Sciences, University of Waikato, Hamilton, New Zealand; ^2^ Department of Psychology, Lund University, Lund, Sweden; ^3^ School of Psychology & Counselling, Open University, Milton Keynes, United Kingdom; ^4^ Independent Scholar, London, United Kingdom

**Keywords:** differences in sex development, intersex, children, young people, disclosure, decision-making, DSD, parents

## Abstract

**Introduction:**

Over the past two decades, there has been a shift from concealing diagnoses of sex development from impacted people to the broad principle of age-appropriate disclosure. This change is consistent with children’s rights and with general shifts towards giving children medical information and involving patients in medical decision-making. The present paper examines how health professionals, young people and caregivers with experience in this area talk about the process of telling children about a diagnosis relating to sex development. The focus is on (i) who is given the role of talking with children and young people about their medical condition and care in the context of a diagnosis relating to sex development and (ii) what strategies seem to work, and what dilemmas are encountered, in engaging children and young people in talk about their condition and healthcare.

**Method:**

Qualitative semi-structured interviews were carried out with 32 health professionals, 28 caregivers and 12 young persons recruited in the UK and Sweden, and thematic analysis was undertaken.

**Results:**

The analysis identifies strategies and dilemmas in communication and a widespread assumption that it is caregivers’ responsibility to talk with children**/**young people about the diagnosis. This assumption creates difficulties for all three parties. This paper raises concern about children**/**young people who, despite a more patient-centred care ethos, are nevertheless growing up with limited opportunities to learn to talk about intersex or differences in sex development with confidence.

**Discussion:**

Learning to talk about this topic is one step towards shared decision-making in healthcare. A case is made for services to take clearer responsibility for developing a protocol for educating children and young people in ways that involve caregivers. Such a process would include relevant medical information as well as opportunities to explore preferred language and meaning and address concerns of living well with bodily differences.

## 1 Introduction

In the past, information about intersex/differences in sex development (DSD) was routinely concealed from impacted people. This was consistent with historical medical practices of concealing information from patients in general and children in particular, and such concealment was widespread across many medical issues. The assumptions that underpinned concealing practices led to shame and stigma being communicated to children growing up with intersex variations. These practices are no longer tenable and recent consensus documents emphasise truth telling as integral to contemporary care standards. For example, the 2018 consensus statement on intersex/DSD states: “explaining the condition in an age-appropriate language to the child and adolescent facilitates acceptance and can help to reduce fear and stigma” (1, p. 419).

Although care documents point to the importance of patient education, with the understanding that truth telling is integral to ethical practice and that knowledge is foundational for health and well-being (1, 2), they fall short of explaining how disclosure to children should take place. References to age or developmental appropriateness in talking to minors are common, but details are sparse on how to implement the recommendations. What needs to be disclosed, by whom, and when, and what factors should caregivers bear in mind as they engage in this complex task? Despite an increasing number of resources developed by advocacy groups in recent years, a UK support group concluded from a recent survey that the vast majority of families, young people and adults actively conceal the diagnosis (3).

A study in the US involving female children with congenital adrenal hyperplasia (CAH) and their caregivers suggests that caregivers do talk to younger and older children about CAH (4). The discrepant observations between this study and the UK survey may reflect methodological differences. Children in the US study recalled having early conversations with caregivers, even if they tended not to recall the specific content. Many said that CAH was out-of-sight and out-of-mind until they had to take medication or were socially restricted by the treatment in some way. The children expressed not wanting CAH to define them. Some caregivers described being “matter-of-fact” about communication (4, p. 676). The research team could not identify a common language amongst caregivers to describe CAH to children. On the whole, caregivers mainly felt that they had not done a good job. The study raises interesting questions about how disclosure might actually take place and suggests that more research on this topic in intersex/DSD might be useful.

Although research dedicated to children’s experiences of receiving explanations about intersex/DSD is sparse, conversations on the topic in other healthcare settings provide useful reference points. A systematic review suggests that the views of young people with a range of long-term conditions are not always adequately taken into account when healthcare decisions are made, causing some young people to experience “anxiety, and feelings of being undervalued and excluded.” (5, p. 1731). Being under-informed leads easily to being under-involved in decision-making. Some researchers have furthermore highlighted a sense of betrayal, mistrust and anger in instances where children have grown up not knowing about their medical condition, being told partial or false information about it, and knowing that there is a secret about them within the family without being allowed to know the nature of the secret (6).

Ongoing dialogue with children about their healthcare, on the other hand, provides a foundation for engaging them in healthcare decisions as they develop. Good communication therefore serves the interests of children as well as adults (7, 8). Conversations in various healthcare settings are no longer about whether or not to tell children about their medical conditions (9) but about how to improve the quality of communication.

A study on caregivers’ experience of talking with their children about sex chromosome conditions (6) identified the following barriers to quality communication: caregivers’ difficulty in understanding the necessary information; the emotionally distressing nature of the condition or circumstances; caregivers’ difficulty in knowing how and when to explain the information to their child; caregivers’ concern about the possibility that the information will distress their child; the language surrounding the condition can present a barrier due to the inaccessibility of medical terminology and the connotations of sex-related terms.

Caregivers across healthcare settings have reported feeling challenged by having to talk openly with their children about sex related topics (10). It would seem that these difficulties are reproduced even in specialist services which are developed to specifically address sex development concerns and even when ethical grounds for honest disclosure to children and young people about their diagnoses relating to sex development are well established (11) and widely endorsed.^1^

The 2018 consensus statement suggests the kinds of topics that should be discussed with children from the age of four upwards, including information about sex and gender, naturally occurring variations, useful vocabulary, puberty, privacy and secrecy, dealing with bullying, and fertility, for example (1). Despite these positive intentions, clinical literature tends not to address discussions with children and young people about the significance of reproductive and sex characteristics or about the experience of bodily difference in the social context. Information is often presented in biological terms, which may draw attention away from the psychosocial implications of living with a diagnosis relating to sex development.

The literacy requirement for understanding and explaining about a diagnosis of sex development is substantial. Clinicians can play a bridging role between caregivers and child (12), but caregivers rarely report feeling supported by health professionals to undertake the task of talking with their children about intersex/DSD with confidence (6, 13). Caregivers have expressed a wish for more guidance from experts (14–17) as well as materials and resources for the ongoing process of communication within the family (6). McCauley (18) sets out an educational approach for clinicians to talk with caregivers and young people. A recent study furthermore shows how a constructive role could be taken by volunteers with variations in sex characteristics in talking with caregivers (19). Some experts have assisted support groups^2^ to produce pre-digested and engaging medical information for caregivers and children. However, these collaborations appear to be based on the premise that caregivers are responsible for relaying expert medical information to the child and are able to take account of the child’s cognitive and emotional capacity.

The present paper is based on an analysis of how young people, caregivers and health professionals respond to the challenges of communicating about intersex/DSD. The questions of interest are: (i) who is given the role of talking with children and young people about their medical condition and care in the context of a diagnosis relating to sex development? (ii) What strategies seem to work, and what dilemmas are encountered, in appropriately engaging children and young people in talk about their condition and healthcare?

## 2 Method

The dataset analysed here consists of semi-structured interviews with 12 young persons with a diagnosis relating to sex development, 28 caregivers with a diagnosed child and 32 specialist health professionals. The recruitment of health professionals took place across 12 hospitals. All health professionals who participated in this research were working within, or in close relationship with multidisciplinary teams (MDTs) specializing in diagnoses relating to sex development in Scotland (n = 6), England (n = 19), or Sweden (n = 7). Health professionals included psychologists (n = 7), endocrinologists (n = 6), urologists (n = 6), gynecologists (n = 4), geneticists (n = 3), nurse specialists (n = 3) and pediatric surgeons (n = 3).

The young people who participated were aged 15 – 26 years (mean age = 21 years) and were all located in the UK. They were recruited *via* a support group (n = 2) or a hospital-based clinic specializing in diagnoses relating to sex development (n = 10). In the hospital, the 23 young people (aged 15-26 years) who attended the clinic during September and October 2013 were approached. All were informed about the study and asked if they wanted to participate. Of these, 13 declined the offer, due to not being interested in talking about their experiences or lack of time to participate in an interview. All young people who participated were assigned female at birth and they identified their diagnoses as including CAH, AIS, gonadal dysgenesis and MRKH.

Caregivers were recruited from Sweden (n = 9) and the UK (n = 19) *via* support groups in 2013 and 2014. The 28 caregivers included 20 mothers and 8 fathers representing 25 families. Each caregiver who participated had at least one child with 46,XX CAH (n = 15) or a difference in sex development (n = 13) such as AIS, gonadal dysgenesis or 5-alpha reductase. One interviewee had three children with diagnoses, four interviewees had two children with diagnoses, and the remaining 23 caregivers had one child with a diagnosis. The children in question were aged between six months and 24 years (mean age: 9.9 years).

Health professionals were interviewed by KR, an academic psychologist. Caregivers and young people were interviewed by TL, a clinical psychologist and researcher who is fluent in Swedish and English. At the time of the interviews, TL was a doctoral researcher supervised by KR and PH. Interviews were audio-recorded and transcribed in full.

The research with health professionals was reviewed and given ethical approval by NHS National Research Ethics Service (reference: 11/LO/0384) and University of Surrey Ethics Committee (reference: EC/2011/68/FAHS). The research with caregivers and young people in the UK was approved by the National Research Ethics Services: NRES Committee London/West London (REC: 11/LL/0385), the Joint Research Office at University College London Hospitals (R&D Project ID: 11/0143) and the Ethics Committee at the University of Surrey (EC/2012/52/FAHS). The research with caregivers in Sweden was approved by the Regional Ethics Committee in Stockholm (2008/1671–31/3).

The analysis presented in this paper is part of the SENS project which has been described further elsewhere (17, 20–24). In order to optimize anonymity, participants were unaware of each other. The health professionals who took part in this study were not necessarily in contact with, or referring to, any of the caregivers or young people who took part in this study and vice versa.

The 12 participating young people in the study were asked when they first found out about their condition, who they found out from, what they found out and how it was communicated. The 28 caregivers were asked if they had spoken to their children, how much their children knew about their condition and how this knowledge was communicated. The 32 health professionals were invited to talk about how they engage with caregivers, children, and young people, how they explain options for intervention and how they talk about decisions to be made. They spoke about what and how information is disclosed to children and young people and by whom.

### 2.1 Analysis

Data relating to talking to children and young people about intersex/DSD and related treatment were identified within the interviews and coded inductively (25). In accordance with thematic analysis, the analytic process involved identifying repeated themes within the data and organizing and describing the data in relation to those themes. In the last step of the thematic analysis, we examined how health professionals, caregivers and young people each positioned themselves in the process of talking about the relevant condition, and read the three groups in relation to one another. Below, we present themes for young people, caregivers and health professionals separately (see **Table 1** for summary of themes).

**Table 1:** Overview of data sources and themes.

Data Source	Themes	Data examples
Children & young people	Figuring things out Always knowing Feeling ambivalent Valuing autonomy	Always knowing: “I always knew … I think I was fourteen when I got told the full diagnosis, I asked for it … when I was at the doctors.” Valuing autonomy: “In an ideal world … The doctor would have asked me, ‘Would you like to talk, would you like me to talk to you or your parents, or both of you together?’ And I would have said, ‘I’d like to talk to you alone.’”
Caregivers	It’s not easy Illness talk Anatomy talk Difference talk	It’s not easy: “I never told [my daughter] properly about her condition. … I didn’t think she needed any more stress.” Difference talk: “I used to … tell her how special she is all the time and how it’s good to be different. … [now she’s] very comfortable now in her own skin and she doesn’t care if she’s different.”
Health professionals	Family secrecy Conflicts and dilemmas Psychological expertise	Family secrecy: “…at the moment she knows nothing about her condition whatsoever…” Conflicts and dilemmas: “if the parents aren’t telling [the child] … what has happened, what does [the child] think she is coming to hospital for?”

## 3 Results

### 3.1 Interviews with young people

Young people who participated in the study described how they found out about their medical condition in three common ways. These were (1) figuring out by piecing things together from the questions adults asked them or said within their hearing; (2) believing they had always known about it but discovering that what they had been told was not true; and (3) being given information when they asked directly. Finally, some young people described avoiding the topic rather than wanting to ask directly. This avoidance is linked with the likelihood of further unwanted medical intervention in at least one instance. Each of these experiences of disclosure is exemplified below.

#### 3.1.1 Figuring things out

Many participants described their interactions with clinicians as only providing clues that there is something wrong with them rather than allowing them to be actively involved in conversations about biomedical processes and rationales for treatment. One young person for example reflected on working out what was happening when interacting with doctors:

doctors asking you questions … you sort of gather that (laughter) there’s something going on. … even as a young kid you work something out (laughter) yeah…. I don’t think it, I was never sat down and explained from A to B.

Some remembered turning points when information suddenly became clearer and their knowledge crystalized. This can happen when the young person initiates searches on the internet or moves to an adult service, for example:

I think when I’d moved … to [the adult clinic] … the first time ever, I was actually given a factsheet about my condition … it was all stuff that I knew but in a better format that was easier to understand. But then maybe if I’d been given that earlier … it would’ve been (laughter) a lot easier.

According to the above participant, children are told something along the way but the pieces of information may not hang together. A different participant gave a very clear view on how health professionals and caregivers should approach communication about a diagnosis with a young person: “I think that … they should be talking with the child very early on.”

#### 3.1.2 Always knowing

Many young people interviewed did not recall the first time their diagnosis was raised. Some said that they had always known and some young people described their caregivers managing this situation well. One young person said:

[It] was never a secret … I can remember going to a lot of hospital appointments when I was little … then had weekly checks for quite a long time … my mum had um, photographs … of me in surgery for example … and I can remember seeing those once in a photo album … Mum was like, “Oh yeah that’s the operation you had when you were a baby.” … she did not go into details … but I wasn’t, I suppose I was never really lied to.

As the above excerpt suggests, whatever adults may or may not say, a child would know that they take pills, have spent time in hospital, have had surgery and are medically monitored. Even when a child or young person recalls these details, they do not necessarily know about the nature of their diagnosis, or what any treatment or monitoring was for. Another participant who described always knowing offers more insight into these questions:

I always knew… ‘cause obviously I’ve got scars … so I knew that they were from an operation … I think as soon as I was old enough … I was told I had like a hernia that had to be removed … in case anyone asked about the scars which I had to say that…. I think I was fourteen when I got told the full diagnosis, I asked for it … when I was at the doctors.

While this young person begins by saying that they “always knew,” it would appear that as a child, they did not know any more than a concealing lie (that a hernia had been removed), until they asked the doctor for information, which they happened to do at the age of 14 years. These observations suggest that as an expression, always knowing is not the same as always understanding one’s own body, treatment and ongoing care plan.

#### 3.1.3 Feeling ambivalent

Telling young people sooner gives them more opportunity to cope with the information, whereas withholding information or delaying communication can lead to hurt, anger and a sense of disempowerment (26). However, the young people in our study acknowledged that, had information been made available to them sooner, they might not have been open to it. Some expressed disinterest in engaging in any depth with the topic in a way not dissimilar to the expressions by young people with CAH in a recent study (4). A participant in our study reflected, “you’re quite young and you don’t want to take it in”. As caregivers may also hesitate to “burden” the child (see below), the mutual ambivalence could mean that communication is delayed or patchy, with each party expecting the other to initiate discussion and interpreting the absence of such initiatives as reflective of not being ready or not wanting to engage.

Caregivers may rely on a child or young person to ask questions, but it is not clear from the current study that children or young people are necessarily comfortable doing this – an observation that resonates with an earlier study (27). In relation to her communication with doctors, a young interviewee in the current study expressed that although she “was comfortable up to a point,” she “didn’t really like discussing anything with the doctors.” The young person said, “I would never ask questions of [doctors]”. Different participants gave different reasons for not wanting to ask questions. One described being in a “bubble,” not wanting to share things or ask questions. She described her concern that asking questions might be interpreted as an invitation for medical intervention and she said that she “didn’t want to ask them about it ‘cause I didn’t want them to do anything about it”. This participant explained that staying in a bubble and not wanting to talk was related to a sense that she had not been allowed to keep things private in her life, or to be in control of her life, because of the imperative to share so much in the course of medical interactions:

Part of me almost feels like (pause) I haven’t been in control of my life … I didn’t have anything personal or private about myself ‘cause you had to share it with everyone.

#### 3.1.4 Valuing autonomy

Children inevitably glean understandings and sense emotions relating to bodily difference and medical interventions. This does not mean that they have gleaned accurate information or that they feel comfortable asking questions about things they have experienced and may not understand. It does not mean that they have any idea how the condition or treatment might impact on them over time. They may be left with unarticulated fears and uncertainties that good communication with adults might address.

When young people reflected on their childhood experiences, they acknowledged that they sometimes did not feel safe to ask questions and sometimes did not feel interested in talking about the topic. Now that they were a little older however, they felt it best that children were told as soon as possible. They suggested being given both verbal and written information. They felt that it was important to be able to choose which adults were involved in conversation with them at a particular time point. For example, an interviewee explained that it would have helped her a lot if she had been asked, by the doctor, whether she wanted her caregivers to be involved in the initial conversation about her diagnosis:

In an ideal world … The doctor would have asked me, “Would you like to talk, would you like me to talk to you or your parents, or both of you together?” And I would have said, “I’d like to talk to you alone.”

### 3.2 Interviews with caregivers

The caregivers who participated in this study reported being in different stages of disclosure. Some of them considered their children too young to understand the information and had not raised the topic; some had talked with their son/daughter and found a receptive audience; and some had tried to raise the topic but had been met with disinterest or resistance. How caregivers experienced the process of disclosure to the child and what they have told the child is best captured under four headings: (i) it’s not easy; (ii) illness talk; (iii) anatomy talk; and (iv) difference talk.

#### 3.2.1 It’s not easy

Most of the caregiver participants in the study expressed that it is not easy to know when and how to talk about diagnosis, healthcare and bodily difference with their child. Caregivers explained that there are many moving parts in terms of timing, content and strategies for talking to children about their health and their difference.

In several instances, caregivers considered their children too young and that “it is still too complicated” for the children to be told. Some anticipated talking to the child when they start school or “when they are older”. One mother felt that her daughter had too many other things to deal with and did not want to burden the child further. This mother reported that this line of reasoning was categorically discredited by a medical expert in front of her child:

I never told [daughter’s name] properly about her condition. … I didn’t think she needed any more stress … She knew she had [diagnosis] but I never told her about all the surgery … Um, then we had an appointment with [surgeon 2] … to see if there was any further surgeries needed and … he said … “So [daughter’s name] do you know all about what’s gone on?” And I said to him, “Hold it a minute, can I speak to you on your own? … I need to explain to you that she’s not aware of certain things.” … And in front of her he said, “What kind of mother doesn’t tell their daughter what’s wrong with her?”

This mother explained how her interpretation of what was in the child’s best interests is very different from that of the medical expert. Caregivers described the need to take into account their children’s capacity and circumstances at the time. Another mother said that they needed to take a day at a time and see how their daughter reacts to forthcoming check-ups and respond to the daughter’s cues then.

Caregivers articulated several reasons why they did not talk more openly with their children. Some caregivers also spoke of being afraid to “bother” their children too early. A father said that he does not know what his daughter thinks about the condition and that he does not want to ask her “[b]ecause I don’t want to start putting thoughts in her head”. Other caregivers said that they have to remember to stop talking over the child’s head and instead start talking to or with their children.

On the whole, caregivers reflected on an ongoing dilemma of whether or not to disclose information about the diagnosis to the child. They felt that in telling their child aspects of the diagnosis, there might be a risk of the child sharing sensitive information with peers before they understand the consequences of this action. Several caregivers said that they would appreciate support from a psychosocial professional to help with this process of talking with children and youth about intersex/DSD.

#### 3.2.2 Illness talk

Caregivers of children with CAH over the age of two often said that they had talked to their children about why they have to take medication. Some said they started as early as possible while others said that they waited for their children to start asking questions. They offered examples of saying to the child “your kidneys do not work,” “your adrenal glands do not work,” “you need to take medicine not to die,” you need to take medication “or else you will be sick,” in order to make taking medicine intelligible. One mother explained that it is usual to focus on the practical issues related to the condition, rather than talking about more complicated details like how adrenal glands work.

Caregivers spoke of needing to find ‘natural’ opportunities to talk about the condition and two opportunities that were repeatedly mentioned were around the time of appointments, and when the child explicitly asks for information. They acknowledged that some topics are more difficult to raise than others. The strategy many caregivers seemed to use was to say enough to enable the child to manage their body and healthcare settings but without giving them detailed explanations of how their body came about or its “difference.” While this kind of illness talk was most prominent among caregivers of children with CAH, some caregivers of children with other variations described similar talk if their children had had surgical interventions and/or needed hormone replacement therapy.

#### 3.2.3 Anatomy talk

One mother said that talking about medication is not that hard, but talking about surgery, genitals and difference is more complicated. When it comes to the 11 children who have had surgery, 9 caregivers of 6 children said that they have talked about surgery with their daughter, with one having to do so after a health professional told the child first, against the mother’s wishes. Four caregivers of the other 3 children expressed feeling a need to talk to their children about surgery but had not done so.

Others had tried to talk more generally about sex, bodies and gender. One mother said her daughter talks about “having a penis” and she tries to help her understand that she has a larger clitoris than most other girls, but that it is still a clitoris. Another mother has tried to talk about genital sensitivity. Finally, one mother expressed trying to talk more about how her child “feels” instead of just focusing on “what is between their legs”.

Some caregivers used support group-meetings as an opportunity to discuss surgery, and some have done so to prepare the child to make the most of the support group meeting. That is, the mother would say to the child that other children have also had surgery, in order to help her own child feel like being “one in a crowd”. Other caregivers talked during a meeting where there was a presentation about surgery and the daughter came in and asked what the presentation was all about. This mother explained to the daughter that she had had surgery – hence the scars. One caregiver said that the child seemed “more ready” to have the discussion after the meeting.

Several caregivers in our study likewise said they only talked “briefly” with their child and that they will wait for “the child to ask them further questions.”

#### 3.2.4 Difference talk

Some caregivers explained how a reason for talking with children is to help children to accept themselves, instead of feeling ashamed about having a body that is different from most others’ bodies. To achieve this goal, caregivers use a variety of strategies. One strategy involves talking about other chronic conditions such as diabetes or allergies to show that differences are common. Some caregivers wanted to get across the idea that the diagnosis could have been so much worse and did this by comparing their child’s diagnosis to other diagnoses. However, some caregivers reported focusing explicitly on the “difference” aspect of intersex/DSD. A mother believed that her daughter is comfortable with being different because of the strategic way she had talked with the daughter:

I used to … tell her how special she is all the time and how it’s good to be different. And … a good thing has come from it because she isn’t a follower, [daughter’s name]’s very comfortable now in her own skin and she doesn’t care if she’s different.

One study on talking with children about sex chromosome conditions provides examples of depathologizing and child-friendly terms that families have developed when talking about their child’s chromosomal variation. This included referring to the diagnosis positively such as “Xtra special” “X-man” and “Super X” (6). One mother in our study said that she got her daughter to watch TV-shows about people with some form of difference to be aware of diversity. Several caregiver participants expressed the hope that being in a support group and connecting with others with the same condition can help their child to deal with difference. However, one of these mothers said that she had tried to be very pedagogical about the body, having books about the body at home, but that her attempts are usually unsuccessful; the daughter thought those books were disgusting.

Our data suggest that caregivers’ experiences of talking with children are diverse, from not wanting to “bother” the child by focusing only on medication to having conversations about the sex anatomy. A range of responses from their children was reported including resistance and indifference. Some caregivers have developed strategies for ensuring that the topics of bodily differences and medical conditions become ongoing live topics in the household. Our interviews with health professionals give more insight into what happens when caregivers do not talk in a timely and consistent manner with their children about their condition.

### 3.3 Interviews with health professionals

While disclosure led by caregivers is now normative in relation to diagnoses of sex development, our data has thus far shown many reasons why caregivers find disclosure risks negative consequences. Clinicians may not be fully aware of these difficulties in parenting. Clinicians interviewed for this study emphasized that children and young people must be told about their diagnosis and its implications: “telling the child is very important, [so] that the child has full information”. Responsibility for telling the child is squarely placed on caregivers. Health professionals repeatedly and categorically said that the appropriate disclosure pathway is from clinician to caregiver to child.

The focus of our analysis here is on what happens when caregivers do not feel able to meet this expectation and timely disclosure has not happened. The pediatricians in the study, who wanted to build a working relationship with the growing child and move forward with treatment, tended to consider themselves as having been put in a difficult situation by uncooperative caregivers who have failed the child.

#### 3.3.1 Family secrecy

According to the accounts of health professionals who participated in our study, the practice ideal enshrined in recent care documents, of informing children and young people, is simply not working for some families. Shame and secrecy persist as significant obstacles. Some children and young people growing up with differences in sex development are being kept just as much in the dark as they would have been in times past. Health professionals said caregivers’ resistance to disclosing to the child was based on: 1) caregivers’ negative or prejudiced views about the medical condition; 2) the idea that no one should be told or the young person’s chance of marriage would be ruined; 3) the view that the child would not understand; 4) the view that the right age to tell a young person fully is 18 years old; 5) fear of telling them. One health professional described caregivers as saying “don’t make me do it”. However, some health professionals indicated that, in their experience, caregivers preferred to be the ones to tell children.

Reflecting on an instance where disclosure had not yet happened, one psychologist said:

… we are currently debating with a family about this and this young lady is probably about eight … but at the moment she knows nothing about her condition whatsoever … she doesn’t know why she takes medicine or anything [because of … ] family secrecy shame anxiety … there is often anxiety from the parents’ point of view that they can almost find themselves to get comfortable telling their child but the worry is well who they are going to tell.

The possibility of the child talking to others is often noted by clinicians and researchers. In the above excerpt, the clinician clearly locates the problems of secrecy, shame and anxiety within the family. It would seem that the MDT is currently working alongside the family and maintaining silence. It is now down to the psychologist to reach a point with the caregivers when the latter are ready to talk to the child.

#### 3.3.2 Conflicts and dilemmas

Health professionals talked about trying to maintain a good relationship with young people even as they have to carry out interventions on young people who do not know what medical condition has led to those interventions. The timing of talking with children seemed especially pertinent in relation to inducing puberty. One pediatric endocrinologist, for example, said:

I think it’s very important to engage the family so that they don’t feel you’re going behind their back … The family have to understand why it’s so important for the child to know. And why honesty is the best policy.

The above excerpt, which is typical of many, suggests that managing the relationship with the family is important, as well as attending to the best interests of the child. The dilemma being addressed here involves a balancing act between what is important for the child and how to maintain a good alliance with a family whose approach to (not) talking with the child does not fit with current healthcare norms around disclosure.

An endocrinologist describes working with a family in the context of puberty induction where the young person concerned still does not know what their diagnosis is. They said, “I am trying to build up some kind of relationship because … we have to start … To induce puberty … I tried for a long time to get them here … it was really hard and the parents came themselves and they didn’t want to take the girl with them.” The researcher asked: “if the parents aren’t telling her … what has happened, what does she think she is coming to hospital for?” To this question the endocrinologist replied: “Exactly that is what I tried to tell the parents.” One strategy that this clinician described trying was to put the parents in contact with parents who had disclosed information, about the same kind of diagnosis, to their daughter.

There are many cases where medical experts gave examples of how caregivers seemed to resist their efforts to move towards information sharing with the child. Rather than wait for the caregivers to decide on timing of disclosure, some clinicians have disclosed information to the young person directly. One clinician talked of having imparted information to a young person they were treating and then being met by the caregivers’ anger because “they thought it was too early to tell her”. In this instance, hormonal treatment had started. The endocrinologist explained:

“…when there is no pubertal development … the child needs to know you are not going to go through your puberty in the normal way.”

The above clinician reflected, “historically I think physicians have been trapped in that situation you know when, is it better for the patient not to know … and today we have decided in general terms that the patient should always know.” Another clinician described:

“I’ve had my hands tied by parents saying, ‘I don’t want the child to know until they’re fifteen, sixteen.’”

#### 3.3.3 Psychological expertise

When there is inadequate disclosure from caregiver to child, a psychologist might be called upon to support caregivers. This resonates with previous work on the role that psychologists play in MDTs to manage challenging emotional situations (24, 28). Psychologists in the present study indicated that it could be a very slow process to encourage caregivers to speak with their child. They spoke of a concern that caregivers might disengage from the service when put under too much pressure. The psychologist interviewees referred to a number of strategies they used to encourage disclosure, including: thinking together with caregivers about the value of being told in a timely manner from the young person’s point of view; preparing caregivers for the fact that the young person would be told anyway; modelling information sharing when talking with caregivers about the child’s diagnosis; role playing the disclosure process to help caregivers rehearse what to say to the child; offering resources and ideas, and putting caregivers in contact with other families where disclosure has already taken place. Some psychologists talked about using joint appointments with medical and psychological input and “encouraging parents” to talk to the child by framing it as information sharing.

## 4 Discussion

Pediatric experts recognize the importance of adults sharing diagnostic and treatment information with children and young people. It is clear from our data and other recent research that disclosure to children and young people happens more now than it used to, but actual practices remain highly variable. Many caregivers appear to have accepted the task delegated to them. However, they do not always feel adequately equipped, supported or prepared to carry it out. Most caregivers in this study expressed that it is not easy to know when and how to talk with their child and this theme is analyzed more fully and presented elsewhere (17). Findings of the present study support those in a previous study where young people raised concerns about receiving information from caregivers who themselves did not have a good enough understanding (27). The young informants and caregivers in our study remind us that disclosure is not a didactic process but one that is negotiated and responsive to dynamic verbal and nonverbal cues.

Some caregivers in the present study indicated that they were waiting for their child to ask questions. This resonates with a study relating to young people with cancer, which showed that sensitive issues (e.g. fertility) are more likely to be discussed if the young person raises that topic themselves (29). However, waiting for a child to ask places that child in a position of responsibility, when they are unlikely to know what questions to ask.

Although keeping patients informed about their condition and their care is a core ethic in service provision, it cannot be assumed that patients in healthcare services are waiting to embrace knowledge and agency. A degree of ambivalence towards engaging with one’s own medical diagnosis in depth is to be expected. Citing social research, intersex scholar Iain Morland suggested that some patients may resist being informed and feel disinclined to actively participate in decision making (30, p. 202).

Clinicians express ongoing concerns about those instances where caregivers do not manage the complex task of talking with their children about diagnosis and treatment. Often, clinicians continue to rely on caregivers relaying expert knowledge to the child, even when this approach does not work well. Research suggests that health professionals themselves do not routinely communicate about sexual and reproductive issues even when guidelines state that this should happen, and young people have reported negative emotional consequences from this omission (12, 29). In the present study, health professionals’ references to being trapped and having their hands tied suggest that clinicians perceive themselves being put in a tricky position by caregivers. This suggests the need to develop an alternative approach, rather than leaving it up to caregivers to relay medical and treatment information to children.

In some instances it may be more appropriate for health professionals to do the explaining with caretakers’ involvement. More flexibility around how these conversations happen and who is involved could open the way for children and young people to be more involved in decisions about what is discussed, by whom and when.

### 4.1 Practice implications

In **Table 2**, we summarize key analytic points and draw out implications for improving communication in healthcare contexts. Our data have led us to raise three questions. First, what needs to be in place to ensure all children and young people are told about their condition and treatment in a constructive way? Second, how can caregivers be supported more consistently to initiate conversation sooner rather than later? Third, what about the wider conversations that need to happen for children and young people to make sense of their bodily and healthcare experiences? The strategies set out in **Table 2** show how the present research begins addressing these questions and resonate with strategies suggested by McCauley (18). In addition, many health professionals and advocacy groups have provided helpful resources^3^ to ease caregivers into holding broader conversations with their children about bodies, bodily differences and relating to the world.

**Table 2:** Analytic points and recommendations.

Participant group	Dilemmas	Strategies
Children & young people	It can appear that information was always available, but that information is not necessarily correct. There can be an expectation that a child or young person will be able to ask questions, but this may be too difficult.	It has sometimes worked well when information is given both verbally and in writing. It would be best if information were available in various formats and at various time points. Some young people would prefer to be asked: who would you prefer to talk with you about this, your parents and/or a health professional?
Caregivers	It can be difficult to raise topics such as sex development, surgery and bodily difference. There may be concerns about children telling others. Children and young people sometimes respond with resistance or indifference.	Consider engaging with support groups or talking with other families. Try talking about bodily difference in ways that are valuing. Use books and television programs about bodily differences as a prompt for conversation.
Health professionals	Caregivers do not always manage to talk with their children about their diagnosis and treatment. Healthcare systems do not necessarily have a good way to deal with this issue. It can be particularly dilemmatic when the child reaches an age where puberty (induction) would normally take place but conversations have not yet begun.	Put families in contact with others who have managed to talk with their children. Put families in contact with support groups. Role-play conversations. Model disclosure when talking with caregivers. Talk with caregivers and children together. Help caregivers to think about where and when they might talk with their children. Offer resources and ideas. Explain why it is important to talk with children in timely ways.
Conclusions and recommendations		
Concluding concerns	Expecting caregivers to relay information from health professional to children does not always work well. There is not yet sufficiently consistent or shared understanding about when in a child’s life conversations about DSD should happen. It is not clear who checks if the information is not relayed to a child, meaning that the historical silence may go unbroken in some instances, despite professional intentions to the contrary.	
Recommendations	Equip community-based health communicators with resources to support families to have timely conversations about diagnosis, treatment, sex development and bodily difference. Ensure that all hospital-based DSD-related interventions (including diagnostic testing, hormonal interventions, surgery and checkups) take place alongside a communication strategy designed to engage children and young people appropriately in talk about their own bodies and healthcare.	

Talking with children about their bodies, about any past, current or potential treatment, and about medical conditions relating to sex development, would ideally be an on-going process that precedes any treatment due to take place from late childhood onwards. The current research suggests that, while appropriate written and verbal information at key stages can help, it is also important to check that children are on track for developing a coherent and affirming narrative about their body in ways that can facilitate the fullest possible level of participation in decision making.

For children and young people to be meaningfully engaged in talk about matters pertinent to their healthcare, these conversations should take place before puberty, and (where this is relevant) should precede puberty induction by enough time for the child to consider what it will mean to take medication that leads to the development of secondary sex characteristics. Moving to a full communication approach would mean that the child is involved in discussion of pros, cons, alternatives, and choices in which they can have a say.

The belief that children will signal readiness to talk about this topic is not well-founded and waiting for a child to ask questions is not a reliable strategy. Health professionals might helpfully address this concern with families. Young people advocate for information to come directly from health professionals. They specifically want to understand what treatments they underwent in childhood and how those treatments might affect their future. Across studies, young people’s expressions run counter to pediatric assumption that it is the caregivers’ role to explain medical information to children (for a review, see: 31).

Carefully timed and tailored conversations intended to bring children and young people more fully into the dialogue about their own healthcare are not a simple task and present dilemmas for caregivers and clinicians alike. Sisk et al. explain, “children should know they will not be lied to, but neither will they be forced into disclosure discussions” (2016, p. 7). These authors make helpful suggestions for the dialogue between health professional and caregiver as shown in **Table 3**. It should not be up to individual clinicians to struggle with their conscience over whether and what to tell a child, or spend numerous consultations trying to persuade caregivers to talk with the child. It will not help for one party to berate the other or override their express request to not talk to the child yet. Anyone attempting this task could make mistakes. There is room for humility and an acceptance of uncertainties around how this works in each case. A default approach to encourage disclosure should be developed and made available for all families where there is a diagnosis relating to sex development. By formulating a default plan for approaching and supporting caregivers to educate the child (and perhaps the wider family and community), the potential for conflict is reduced. Psychosocial expertise can contribute to such a plan, which would also benefit from peer support.

**Table 3:** Strategic communication approach for health professionals (Adapted from 9).

Strategic approach	Key steps for health professionals
Listening to all parties	Listen to the caregivers and child, allowing them to guide the discussion. Find out from caregivers what they think their child already knows or understands. Check again what are the caregivers’ and the children’s preferences for communication, as they may change over time.
Offering guidance and support	Give caregivers guidance on how to raise the topic with their child. Reinforce all caregivers’ sense that they are a “good parent.”
Addressing challenges	If caregivers are not in favour of disclosure to their children, it might help to use “shuttle diplomacy.” This approach ensures that all parties are heard and opens up for better understanding of the caregivers’ beliefs, understanding and fears.

### 4.2 Conclusion

This study examines health-related communication between adults and children/young people. Such communication is necessary for young people to play a meaningful role in decision-making about their own healthcare, but making this communication work well for all the individuals and families concerned is far from easy. The present study draws data from young people, caregivers and health professionals, allowing an analysis that triangulates three perspectives on the complex situation of healthcare communication in intersex/DSD. The triangulation enables better understanding of the norms at play and the persistent dilemmas of expecting caregivers to relay expert information to the child.

Our data offer insight into how there is sometimes only partial or inaccurate disclosure to children and young people. Talking about this topic with children and young people is a complex process for which caregivers should have access to a variety of resources, including community-based resources and health professionals with specific expertise in communication about emotionally challenging topics. A psychologically informed service strategy for communication entails a collective understanding of disclosure as a process that moves back and forth in response to dynamic verbal and nonverbal cues. This process is unpredictable and challenging for the actors involved but service providers are responsible for supporting all parties to meet the challenges. Such a service strategy recognizes that imperfections are to be expected, and that humility and forgiveness are valuable virtues to cultivate in the process.

We urge experts involved in writing future care documents to discourage services from abdicating the responsibility of health-related communication to caregivers. Caregivers are laypersons with varying degrees of experience and confidence in comprehending and discussing complex medical information, and it is part of responsible service provision to ensure that children and young people can make sense of health care processes in order to participate in decision making.

## Strengths and limitations of this study

This study explores the perspectives of young people, caregivers and health professionals on communicating about intersex/DSD. A strength of the study is that it draws from data gathered in different regions and national contexts, thus providing a picture that is not idiosyncratic to one service or region. The biggest drawback of our research is that caregivers who struggled the most to talk to their child about intersex/DSD, such as those described by our 32 clinician interviewees, and those who had disengaged from services rather than having to disclose to the child, would not have participated in the study and remain unheard. It is their experiences that beg our understanding the most. A further limitation of the study is that we do not have data on the specific diagnostic timing for each family involved. It is not possible to develop interpretations relating to the age at diagnosis or age at disclosure to the child. A more fine-grained analysis based on age data would make it possible to draw specific conclusions about talking with children who were diagnosed as infants and talking with children and young people who were diagnosed later.

## Data availability statement

The datasets presented in this article are not readily available because the data are sensitive and not easily anonymized. Requests to access the datasets should be directed to kroen@waikato.ac.nz.

## Ethics statement

This research was approved by the UK National Research Ethics Services (REC: 11/LL/0385 and 11/LO/0384); the Joint Research Office at University College London Hospitals (R&D Project ID: 11/0143) and the Ethics Committee at the University of Surrey (EC/2012/52/FAHS and EC/2011/68/FAHS). Written informed consent to participate in this study was provided by the participants.

## Author contributions

All authors were responsible for conceptualizing the study. KR conducted the interviews with health professionals and the initial processing and analysis of the data. TL conducted the interviews with young people and caregivers and the initial processing and analysis of the data. All authors contributed to the article and approved the submitted version.

## Footnotes

1 Authors who write about the grounds for honest disclosure to children have often cited the United Nations Convention on the Rights of the Child (article 12): “*State parties shall assure to the child who is capable of forming his or her own views the right to express those views freely in all matters affecting the child, the views of the child being given due weight in accordance with the age and maturity of the child.”* http://www.ohchr.org/en/professionalinterest/pages/crc.aspx United Nations (1989) *Children act.* Respecting the rights of the child involves giving children the opportunity to participate in age-appropriate ways in decisions about their treatment. This would require the child to be given accurate and complete information about their body and diagnosis.

2 For example, dsdfamiles resources such as “Top tips for talking about differences of sex development” https://DSDfamilies.org/application/files/4115/3780/1476/Top_Tips_for_Talking.pdf

3 Resources have been developed by dsdfamilies (https://dsdfamilies.org/resources) and InterACT (https://interactadvocates.org/resources/intersex-resource-topics/intersex-health-and-sex-education-in-schools/), for example. There is also a handbook for caregivers (https://dsdguidelines.org/) and guidelines for psychologists (https://www.bps.org.uk/node/2585).
